# Increases of M2a macrophages and fibrosis in aging muscle are influenced by bone marrow aging and negatively regulated by muscle-derived nitric oxide

**DOI:** 10.1111/acel.12350

**Published:** 2015-05-25

**Authors:** Ying Wang, Michelle Wehling-Henricks, Giuseppina Samengo, James G Tidball

**Affiliations:** 1 Molecular, Cellular & Integrative Physiology Program, University of CaliforniaLos Angeles, CA, 90095, USA; 2 Department of Integrative Biology and Physiology, University of CaliforniaLos Angeles, CA, 90095, USA; 3 Department of Pathology and Laboratory Medicine, David Geffen School of Medicine at UCLA, University of CaliforniaLos Angeles, CA, 90095, USA

**Keywords:** aging, inflammation, mouse, skeletal muscle, fibrosis, macrophage, nitric oxide synthase

## Abstract

Muscle aging is associated with changes in myeloid cell phenotype that may influence age-related changes in muscle structure. We tested whether preventing age-related reductions in muscle neuronal nitric oxide synthase (nNOS) would obviate age-related changes in myeloid cells in muscle. Our findings show that muscle aging is associated with elevations of anti-inflammatory M2a macrophages that can increase muscle fibrosis. Expression of a muscle-specific nNOS transgene in mice prevented age-related increases in M2a macrophages. Transgene expression also reduced expression of collagens and decreased muscle fibrosis. The nNOS transgene prevented age-related increases in arginase-1 but did not influence TGFβ expression, indicating that the transgene may prevent age-related muscle fibrosis by inhibiting the arginase-dependent profibrotic pathway. Although aged satellite cells or fibro-adipogenic precursor (FAPs) cells also promote fibrosis, transgene expression had no effect on the expression of key signaling molecules that regulate fibrogenic activity of those cells. Finally, we tested whether increases in M2a macrophages and the associated increase in fibrosis were attributable to aging of myeloid lineage cells. Young bone marrow cells (BMCs) were transplanted into young or old mice, and muscles were collected 8 months later. Muscles of young mice receiving young BMCs showed no effect on M2a macrophage number or collagen accumulation compared to age-matched, nontransplanted controls. However, muscles of old mice receiving young BMCs showed fewer M2a macrophages and less accumulation of collagen. Thus, the age-related increase in M2a macrophages in aging muscle and the associated muscle fibrosis are determined in part by the age of bone marrow cells.

## Introduction

Aging muscle undergoes a shift in the balance between myogenic potential and fibrogenic activity so that senescent muscle suffers from a reduced capacity to repair and regenerate as it becomes increasingly fibrotic. Over time, the shift can lead to substantial accumulations of connective tissue. For example, recent findings show that the concentration of collagen in the muscles of old mice is nearly twice the concentration in young mice, corresponding to a twofold increase in muscle stiffness (Alnaqeeb *et al*., 1984; Wood *et al*., 2014). At least some of the increased fibrosis is caused by increased collagen expression in muscle (Babraj *et al*., 2005) although reductions in collagen turnover in muscle also occur (Pattison *et al*., 2003). Concurrent with this increase in fibrosis, the regenerative capacity in muscle declines, which may reflect a reduction in the numbers of Pax7+ myogenic cells in the muscle (satellite cells) that are necessary for continued myogenesis (Shefer *et al*., 2006), as well as a reduction in the myogenic capacity of aged satellite cells (Schultz & Lipton, 1982; Chakravarthy *et al*., 2000).

Much is unknown concerning the mechanisms that drive senescent muscle toward fibrosis, but recent findings concerning fibrotic processes in dystrophic muscle or in wild-type muscle that has experienced acute injury indicate that the immune system can play important roles in regulating the balance between myogenesis and fibrosis. This has been clearly demonstrated in association with chronic muscle injuries in which muscle inflammation can persist for months or years. In Duchenne muscular dystrophy (DMD) or the *mdx* mouse model of DMD muscle, inflammation continues from the early onset of the pathology through the entire lifetime of the afflicted (Wehling-Henricks *et al*., 2010). In this case, macrophages comprise the most prevalent leukocyte population (Wehling *et al*., 2001). A subpopulation of anti-inflammatory, M2a macrophages is present at highest numbers through stages of the disease when fibrosis is most extensive, and they are primarily responsible for the shift toward a more fibrotic state (Villalta *et al*., 2009). Typically, macrophages are activated to the M2 phenotype by the Th2 cytokine interleukin-4 (IL-4) or IL-13. However, during skeletal muscle inflammation, IL-10 is required for their activation to the M2a phenotype, which is characterized by elevated expression of CD163 and arginase (Villalta *et al*., 2011; Deng *et al*., 2012). M2a macrophages then promote muscle fibrosis by arginase-mediated hydrolysis of arginine that drives the production of ornithine that is then metabolized to produce proline required for collagen production (Wehling-Henricks *et al*., 2010). Ablation of arginase expression in *mdx* mice greatly reduces the pathological fibrosis of most dystrophic muscles (Wehling-Henricks *et al*., 2010).

Studies of the response of muscle to injury by toxin injection have indicated that other leukocyte populations can influence the balance between muscle regeneration and fibrosis. For example, toxin-injured muscle experiences invasion by SiglecF+ eosinophils that can promote muscle repair (Heredia *et al*., 2013). Those intramuscular eosinophils release IL-4 that activates fibroadipogenic progenitor cells (FAPs) in the muscle to increase their phagocytic removal of cellular debris and lead to more rapid regeneration (Heredia *et al*., 2013). Other studies have shown that muscle injury by toxin injection causes a clonal expansion of anti-inflammatory, FoxP3+ regulatory T cells (Tregs) that increase muscle regeneration while reducing fibrosis (Burzyn *et al*., 2013). Thus, these studies show that leukocytes invading young muscle following acute injury promote regeneration and reduce fibrosis.

The amplified, profibrotic inflammatory response in injured or diseased muscle can be exacerbated by the loss of neuronal nitric oxide synthase (nNOS) from muscle. Nitric oxide (NO) generated by muscle nNOS serves many regulatory roles, but in the context of muscle inflammation, it plays a role in inhibiting extravasation of leukocytes into the damaged tissue (Wehling *et al*., 2001). Because of the absence of nNOS from dystrophic muscle, inflammation is amplified and persistent which contributes to muscle fibrosis (Wehling-Henricks *et al*., 2010). However, muscle-derived NO can also activate satellite cells, which are a population of muscle-specific stem cells that reside in fully differentiated muscle (Anderson, 2000; Tatsumi *et al*., 2002). Satellite cell activation is required for normal muscle regeneration and growth (von Maltzahn *et al*., 2013). Thus, loss of nNOS from dystrophic muscle shifts the myogenic/fibrotic balance toward fibrosis by loss of normal NO modulation of leukocytes and satellite cells.

Skeletal muscle aging also causes large reductions in the expression of nNOS (Richmonds *et al*., 1999; Samengo *et al*., 2012) that accompany the increase in fibrosis and the reduction in regenerative capacity experienced during muscle senescence. Thus, it is feasible that the age-related decrease in muscle nNOS expression contributes to an increase in the numbers and activation of leukocytes that promote muscle fibrosis while also leading to a reduction in the numbers of satellite cells, which would reduce the regenerative capacity of aging muscle. We test that hypothesis in the present investigation by examining the effects of expressing a muscle-specific nNOS transgene on the numbers and phenotype of leukocyte populations in the muscle, the occurrence of fibrosis, and the prevalence of satellite cells in aging muscle. We also test whether age-related increases in macrophage populations in muscle are attributable to the age of the hematopoietic stem cell population from which they are derived, or reflect the age of the muscle in which they reside by performing heterochronic bone marrow transplantations (BMT) between young and old mice and analyzing the effects of those transplantations on muscle macrophage phenotype and fibrosis in old muscle. Collectively, our data show that M2a macrophages in muscle increase with aging in association with increased fibrosis, and we find that preventing the reduction in nNOS expression in aging muscle prevents age-related changes in muscle macrophages and fibrosis, without affecting the prevalence of satellite cells. Our findings also show that the shift of muscle macrophages to an M2a phenotype is strongly influenced by the age of the hematopoietic cells from which they are derived.

## Results

### Expression of an nNOS transgene in skeletal muscle prevented age-related increases in pro-inflammatory cytokine expression and increases in muscle macrophage populations

**Fig 1:**
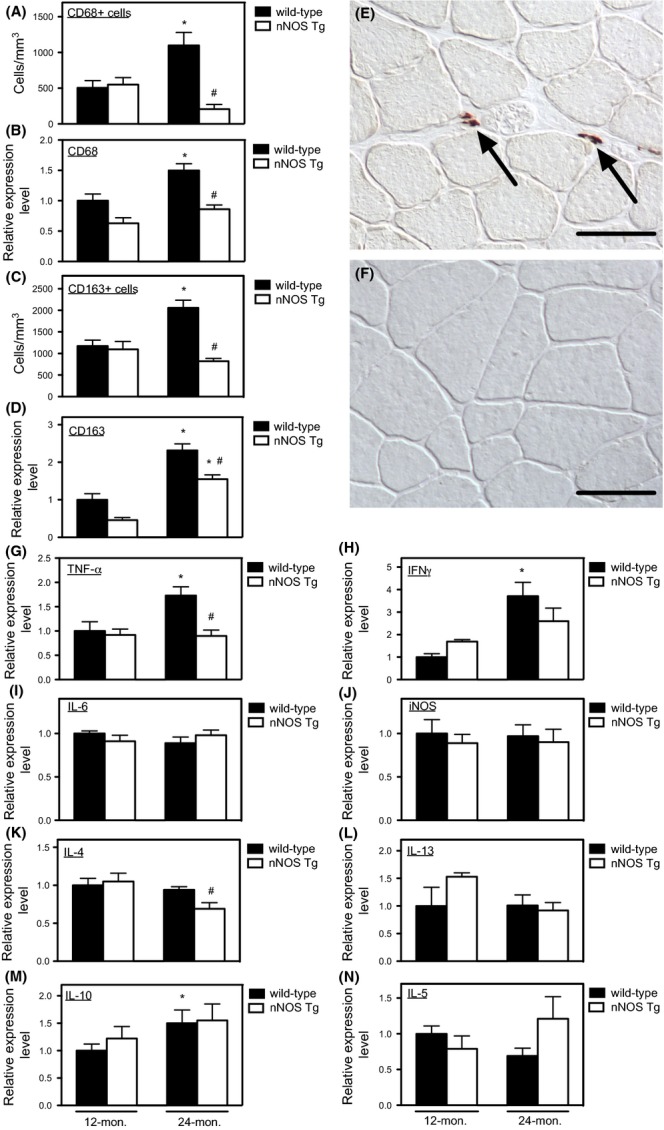
Muscle-derived NO prevents age-related increases of intramuscular macrophages and elevated expression of Th1 cytokines in muscle. (A–D) Expression of a muscle-specific nNOS transgene prevented the age-related increases in: (A) CD68+ macrophage numbers; (B) CD68 mRNA levels assayed by QPCR; (C) CD163+ M2a macrophage numbers; (D) CD163 mRNA levels assayed by QPCR. (E) Immunohistochemistry showing distribution of M2a macrophages labeled with anti-CD163 (arrows) in the thickened perimysium and epimysium of 24-month-old, wild-type quadriceps. (F) Anti-CD163 immunohistochemistry revealed no M2a macrophages in this region of nNOS transgenic quadriceps muscle where the thickness of the perimysium and epimysium resembles 12-month-old wild-type mice. Bars = 40 μm. QPCR data showing increases in the mRNA for Th1 cytokines TNFα (panel G) and IFNγ (panel H) occurred in muscles during aging, but the increases were prevented by transgene expression. Other mRNAs associated with M1 macrophages did not increase with aging and were not affected by transgene expression (panel I, IL-6; panel J, inducible NOS [iNOS]). Expression of mRNA for Th2 cytokines in muscle was differentially affected by aging and transgene expression: (K) IL-4 mRNA did not increase with aging, although transgene expression reduced its concentration in 24-month-old muscle; (L) Neither aging or transgene expression affected IL-13 expression. (M) Aging increased IL-10 expression, but the increase was unaffected by transgene expression; (N) Neither aging nor transgene expression affected IL-5 expression. *N* = 5 for all data sets. *indicates significant difference at *P* < 0.05 compared to same genotype at 12 months. ^#^Indicates significant difference at *P* < 0.05 compared to wild-type at same age.

Previous investigators have reported increases in either the expression of pro-inflammatory or anti-inflammatory cytokines in aging muscle (Peake *et al*., 2010). Our QPCR analysis of hind-limb muscles from adult (12-month-old) and aged (24-month-old) mice showed that aging is associated with increased expression of pro-inflammatory and anti-inflammatory cytokines, although the magnitude of increase varied between muscles (Table1). Th1 cytokines IL-1β, TNFα, and IFNγ were significantly elevated in all senescent muscles analyzed, with the exception of no increase in IL-1β expression in old soleus muscle. However, IL-10 expression was also significantly elevated in old muscle, accompanying the increased expression of the M2a macrophage phenotypic marker, CD163. Quantitation of CD68+ and CD163+ macrophages showed approximately a twofold increase in CD68+ and CD163+ macrophages in wild-type, 24-month-old muscle compared to 12-month-old muscle, in accord with changes in CD68 and CD163 mRNA levels (Fig.1A–D). Macrophages were primarily located in perimysium in all treatment groups (Fig.1E,F). Although the histological data indicated that elevations of CD68+ and CD163+ macrophage populations in aging muscle were prevented by nNOS transgene expression (Fig.1A,C), QPCR data showed that transgene expression prevented the age-related increase in transcripts associated with M1 macrophages (TNFα and IFNγ; Fig.1G,H), but did not affect the elevation of IL-10, which can be expressed by M2 macrophages (Fig.1M). The lack of concordance between the reduction in M2 macrophages in transgenic, 24-month-old muscle and no reduction in IL-10 expression indicates that M2 macrophages are not the primary source of IL-10 expression in old muscle. Other transcripts associated with the M1 phenotype (IL-6, iNOS) or M2 phenotype (IL-4, IL-5, IL-13) were also unaffected by aging (Fig.1 I–N).

**Table 1:** Relative expression levels of inflammation-associated genes in 24-month-old muscles assessed by QPCR. Values shown are expressed relative to the expression levels in 12-month-old wild-type muscles in which the Ct values are normalized to empirically validated reference genes and the log transformation of the normalized mean for the group is adjusted to 1 unit. Standard errors of the mean are shown parenthetically. *N* = 5 for each group

Muscles	Transcripts									
	CD68	IL-1β	TNFα	IFNγ	IL-6	CD163	IL-4	IL-5	IL-10	IL-13
Quadriceps	1.5 (0.11)*	2.74 (0.28)§	1.73 (0.18)*	3.71 (0.61)#	0.89 (0.07)	2.18 (0.12)#	0.94 (0.04)	0.69 (0.11)	1.50 (0.10)*	1.06 (0.21)
Soleus	2.55 (0.18)§	1.07 (0.19)	3.24 (0.35)§	3.08 (0.77)*	0.82 (0.12)	2.32 (0.17)§	1.48 (0.24)	1.16 (0.13)	11.66 (0.6)§	1.52 (0.33)
Tibialis ant.	1.64 (0.07)#	1.72 (0.14)#	2.35 (0.13)§	4.21 (0.25)§	0.83 (0.08)	1.66 (0.04)#	1.34 (0.15)	1.18 (0.03)#	7.96 (1.14)§	ND

* Unpaired *t*-test; *P* < 0.05.

# Unpaired *t*-test; *P* < 0.01.

§ Unpaired *t*-test; *P* < 0.001.

### Expression of an nNOS transgene in skeletal muscle prevented age-related increases in muscle fibrosis

**Fig 2:**
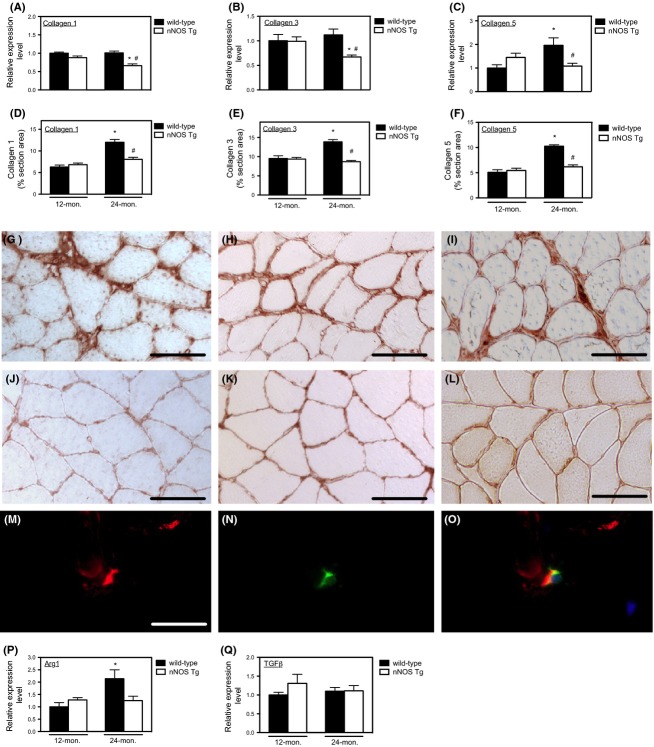
Increases in connective tissue accumulation in aging muscle coincided with elevations of arginase expression and were prevented by nNOS transgene expression. (A–C) QPCR data show that nNOS transgene expression reduced mRNA encoding collagen types I, III, and V in 24-month-old quadriceps. (D–F) The volume fractions of muscle tissue occupied by collagen types I, III, and V assayed by stereology of muscle cross sections showed that each connective tissue component accumulates in muscle between 12 and 24 months of age, but the accumulation was prevented by transgene expression. (G–I) Representative cross sections of 24-month-old, wild-type quadriceps muscles labeled with antibodies to collagen type I (panel G), type III (panel H), or type V (panel I). Bar = 50 μm. (J–L) Representative cross sections of 24-month-old, nNOS transgenic quadriceps muscles labeled with antibodies to collagen type I (panel J), type III (panel K), or type V (panel L) showing reduction in thickness of perimysium and epimysium in transgenic muscles compared to wild-type. (M–O) Immunohistochemistry, double-labeling for CD163 and arginase. Cross sections of 24-month-old wild-type quadriceps muscle labeled with anti-arginase-1 (red; panel M) and anti-CD163 (green; panel N) and the images merged (yellow; panel O) showed that all CD163+ cells expressed arginase-1. (P) QPCR analysis of arginase-1 expression in quadriceps showed that the increase in arginase-1 expression with aging and the prevention of elevated arginase-1 expression in nNOS transgenic muscles mirrored changes in CD163 expression. (Q) QPCR analysis of TGFβ expression showed that there was no effect of aging or nNOS transgene expression on TGFβ levels in muscle. *N* = 5 for all data sets. *Indicates significant difference at *P* < 0.05 compared to same genotype at 12 months. ^#^Indicates significant difference at *P* < 0.05 compared to wild-type at same age.

Because earlier studies showed that loss of nNOS expression in muscle led to greater numbers of macrophages in muscle (Wehling *et al*., 2001; Nguyen & Tidball, 2003) and demonstrated that prolonged elevation of M2a macrophage populations in diseased muscle was associated with increased fibrosis (Wehling-Henricks *et al*., 2010), we assayed whether the NO-mediated reduction in M2a macrophage numbers in aging muscle influenced fibrosis. Expression of the nNOS transgene reduced the level of transcripts encoding collagen type I, collagen type III, and collagen type V in 24-month-old muscle (Fig.2A–C). Furthermore, expression of the transgene prevented the age-related increase in the volume fraction of muscle that was comprised of each collagen type (Fig.2D–F) and reduced the thickness of epimysial and perimysial connective tissue that was comprised of collagen types I, III, or V (Fig.2G–L).

The association between reductions in M2a macrophage populations and the reductions in age-related muscle fibrosis suggested that M2a macrophages could promote fibrosis in aging muscle. Because previous studies showed that M2a macrophages can drive muscle fibrosis via arginase-mediated metabolism (Wehling-Henricks *et al*., 2010), we assayed for arginase expression in aged muscle and found that CD163+ M2a macrophages in old muscle express arginase-1 (Fig.2M–O). Furthermore, we found that nNOS transgene expression reduced arginase-1 expression in old muscle (Fig.2P) by a magnitude that resembled the reduction of CD163+ M2 macrophage populations caused by transgene expression (Fig.1C). Although M2 macrophages can also express TGFβ, which is a pro-fibrotic cytokine, we observed no age-related increase in TGFβ expression and nNOS transgene expression did not affect TGFβ expression (Fig.2Q).

### Expression of an nNOS transgene affected Vangl2 and Pax7 expression in adult, but not aged, muscle

**Fig 3:**
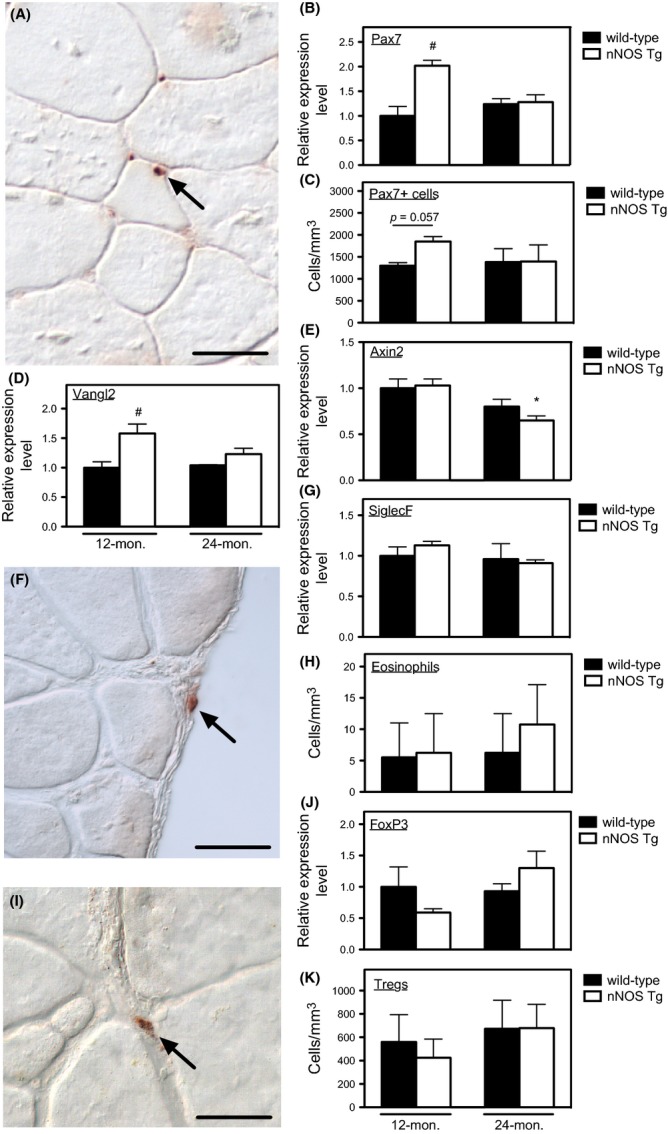
Muscle aging is not associated with changes in Vangl2 or axin-2 expression or changes in satellite cell, eosinophil, or Treg numbers. (A) Representative image of Pax7+ satellite cells (red) at the surface of 24-month-old, wild-type muscle fibers. Bar = 25 μm. (B) QPCR analysis showed that aging did not affect Pax7 expression between 12 and 24 months. Expression of the nNOS transgene increased Pax7 expression at 12 months of age but not at 24 months. (C) Stereological counts for Pax7+ cells showed that aging did not affect satellite cell numbers between 12 and 24 months. Expression of the nNOS transgene caused a strong trend for increased satellite cell numbers at 12 months of age but not at 24 months. (D) QPCR analysis showed that aging did not affect Vangl2 expression between 12 and 24 months. Expression of the nNOS transgene increased Vangl2 expression at 12 months of age but not at 24 months, mirroring changes in Pax7 expression. (E) QPCR analysis showed that aging did not affect axin-2 expression between 12 and 24 months. (F) Immunohistochemistry for eosinophils using anti-MBP on a section of 24-month-old, wild-type quadriceps. Most eosinophils (arrow) were observed in the epimysium surrounding the muscle, rather than in the parenchyma. Bar = 25 μm. (G) QPCR analysis for SiglecF indicated that there was no change in eosinophil presence during muscle aging or as a consequence of nNOS transgene expression. (H) Stereological counts for MBP+ cells show that neither nNOS transgene expression nor aging affected eosinophil numbers between 12 and 24 months. (I) Immunohistochemistry for Tregs (arrow) using anti-FoxP3 on a section of 24-month-old wild-type quadriceps. Bar = 25 μm. (J) QPCR analysis for FoxP3 indicated that there was no change in Treg presence during muscle aging or as a consequence of nNOS transgene expression. (K) Stereological counts for FoxP3+ cells showed that neither nNOS transgene expression nor aging affected Treg numbers between 12 and 24 months. *N* = 5 for all data sets. *Indicates significant difference at *P* < 0.05 compared to same genotype at 12 months. ^#^Indicates significant difference at *P* < 0.05 compared to wild-type at same age.

We further tested whether preventing age-related reductions in muscle nNOS expression affected expression of Vangl2 or Pax7 because previous investigations showed that NO can increase numbers of Pax7+ satellite cells via a Vangl2-dependent pathway that required increased transcription of Vangl2 mRNA (Buono *et al*., 2012). Consistent with those previous reports, we observed that nNOS transgene expression caused significant elevations of Vangl2 and Pax7 expression in adult skeletal muscle and a strong trend for increased numbers of satellite cells (Fig.3A–D). However, transgene expression did not affect Pax7 expression, Vangl2 expression, or satellite cell numbers in aged muscle, indicating that the NO-Vangl2-cGMP pathway for expanding satellite cell populations is not activated by elevated nNOS expression in old muscle. We also assayed whether the rescue of aged muscle from fibrosis reflected a reduction in satellite cell switching to a profibrotic phenotype via activation of Wnt signaling characterized by elevated expression of axin-2 mRNA (Brack *et al*., 2007), but we did not observe elevated axin-2 expression in aged muscles analyzed in our investigation (Fig.3E).

### Expression of an nNOS transgene did not influence eosinophil or Treg numbers in aging muscle

Because elevation in NO production by diseased muscle can reduce numbers of multiple lymphoid and myeloid cell populations other than macrophages in muscle and other leukocytes such as eosinophils (Heredia *et al*., 2013) and regulatory T cells (Tregs; Burzyn *et al*., 2013) can influence muscle regeneration and fibrosis following acute injury or disease, we tested whether transgene expression affected numbers of eosinophils or Tregs in aging muscle. We observed that eosinophils were present in aged, wild-type muscle but only in extremely low numbers, and their numbers did not change with aging and transgene expression had no affect on their frequency in adult or aged muscles (Fig.3F–H). Tregs were more prevalent than eosinophils in adult and aged muscles, but their numbers were not significantly affected by age or transgene expression (Fig.3I–K), indicating that the rescue from fibrosis did not result from NO effects on Treg numbers.

### Transplantation of young bone marrow cells into adult mice prevented the age-related increase in M2a macrophages in muscle

**Fig 4:**
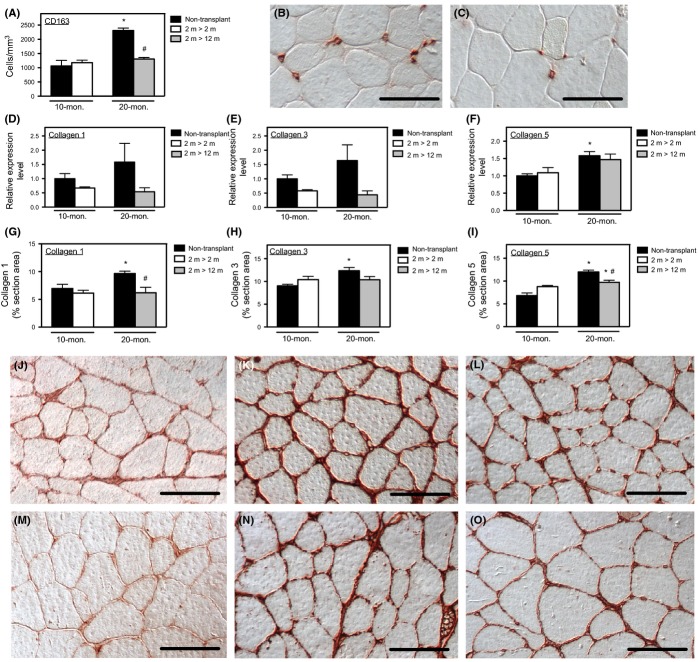
Effects of heterochronic BMT on muscle fibrosis and inflammation. (A) Anti-CD163 immunohistochemistry showed intramuscular M2a macrophages increased in number significantly between 10 and 20 months of age, but the increase did not occur in 20-month-old mice that had received transplantation of 2-month-old BMCs when the recipients were 12 months of age. (B) Representative anti-CD163 immunohistochemistry of M2a macrophages in quadriceps muscle of 20-month-old, nontransplanted mouse. Bar = 40 μm. (C) Representative anti-CD163 immunohistochemistry of M2a macrophages in quadriceps muscle of 20-month-old mouse that received transplantation of 2-month-old BMCs. Bar = 40 μm. (D–E) QPCR for mRNA for collagens in muscle showed no significant differences in the expression of collagen type I or type III between 10 and 20 months of age between any of the treatment groups. (F) QPCR for collagen type V showed that the significant increase in collagen V expression in muscle between 10 and 20 months of age was not affected by transplantation of 2-month-old BMCs. (G–I) The volume fractions of muscle tissue occupied by collagen types I, III, and V assayed by stereology of muscle cross sections showed that 20-month-old, nontransplanted mice had significantly larger volume fraction of the muscle occupied by collagen types I, III, or V than occurred in 10-month-old nontransplanted mice. Transplantation of 2-month-old BMCs prevented the age-associated increase in collagen type I in muscle (panel G), did not affect the increase in collagen type 3 (panel H), and reduced, but did not prevent, the increase in collagen V in 20-month-old mice. *N* = 5 for all groups. *Indicates significant difference at *P* < 0.05 compared to 10-month-old mice receiving same treatment. ^#^Indicates significant difference at *P* < 0.05 compared to nontransplanted mice at same age. J–K: Representative images of cross sections of quadriceps from 20-month-old, nontransplanted mice (panels J–L) or 20-month-old mice receiving transplantation of 2-month-old BMCs (panel M–O) after labeling with anticollagen type I (panels J and M), type III (panels K and N), and type V (panels L and O), showing a decreased volume fraction of muscle occupied by collagens type I and type V in mice receiving BMT from young donors. Bars = 50 μm.

The increase in CD163+ cells in aging muscle could reflect either the influence of aging muscle on macrophage phenotype or reflect age-related changes in the differentiation of hematopoietic lineage cells that are independent of influences from the host tissue. We tested whether muscle macrophage phenotype was determined by the age of the host muscle or the age of the marrow from which the cells were derived by transplanting young bone marrow cells (BMCs; 2-month-old donors) into young mice (2-month-old) or adult mice (12-month-old) and then assaying muscle macrophage phenotype and muscle fibrosis 8 months later. Levels of chimerism achieved 8 months after BMT did not differ between young transplant recipients (circulating leukocytes donor-derived: 95.0%; SEM = 1.9; *N* = 5) or old recipients (circulating leukocytes donor-derived: 97.6%; SEM = 0.28; *N* = 4). Muscles of mice that were not transplant recipients showed increases in M2a macrophage numbers (Fig.4A) that were similar to the increases that occurred between 12 and 24 months of age (Fig.1C). Although 10-month-old mice that received BMT at 2 months of age showed numbers of M2a macrophages in muscle that were identical to the numbers that occurred in nontransplanted, 10-month-old mice, transplantation of 2-month-old BMCs into 12-month-old mice prevented the increase in intramuscular M2a macrophages that occurs during aging to 20 months of age. The muscles of 20-month-old transplant recipients and 20-month-old nontransplanted recipients both showed similar distributions of M2a macrophages in the perimysium and endomysium (Fig.4B,C). These findings support the view that the age of the hematopoietic compartment and not the age of the muscle is primarily important in determining the increase of M2a macrophages in aging muscle.

### Transplantation of young bone marrow cells into adult mice reduced connective tissue accumulation in old muscle

Because experimental reductions in M2a macrophages in aging muscle caused coinciding reductions in muscle fibrosis (Figs1C, 2D–F), we tested whether the reduction in M2a macrophages in 20-month-old mice that received young bone marrow showed similarly reduced levels of fibrosis. As observed between 12- and 24-month-old, wild-type mice (Fig.2A–C), expression of collagens type I and III did not increase significantly between 10 and 20 months, although expression of collagen type V showed a significant increase (Fig.4D–F). However, collagen type V expression was identical in 20-month-old mice that received transplantation of young BMCs and in 20-month-old, nontransplanted mice, indicating that aging of the hematopoietic compartment did not influence collagen type V expression (Fig.4F).

Similar to our findings in comparing accumulation of connective tissue proteins in aging muscle between 12 and 24 months of age (Fig.2D–E), we found that the volume fraction of muscle occupied by collagen type I, type III, and type V increased between 10 and 20 months of age (Fig.4G–O). We also observed that transplantation of young BMCs into adult muscle prevented the age-related accumulation of collagen type I and type III and significantly reduced the accumulation of collagen type V during aging (Fig.4G–I). For each collagen type, accumulation primarily occurred in the perimysial and endomysial spaces (Fig.4J–O). These findings indicate that the aging of the hematopoietic compartment contributes significantly to the accumulation of connective tissue in aging muscle.

## Discussion

**Fig 5:**
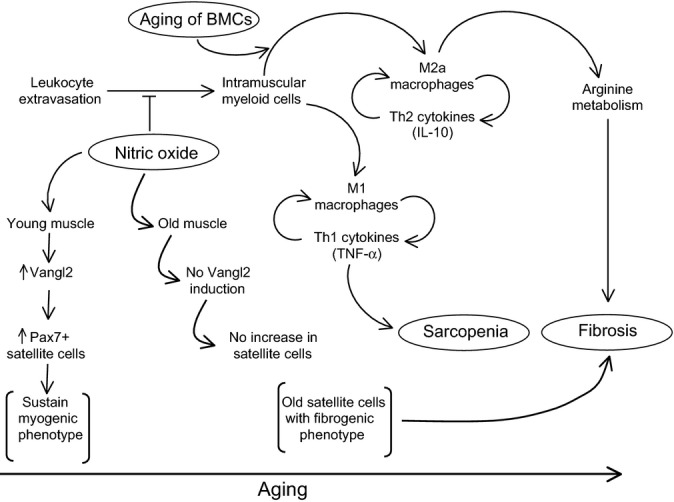
Summary diagram that illustrates potential mechanisms through which muscle-derived NO and bone marrow-derived cells can influence sarcopenia and muscle fibrosis that occurs during aging. In young muscle, relatively high levels of nitric oxide are capable of (i) increasing satellite cell numbers through increased, Vangl2-mediated signaling (Anderson, 2000; Buono *et al*., 2012) and (ii) inhibiting extravasation of leukocytes into the muscle. Age-related loss of muscle nNOS is associated with an increase in intramuscular leukocytes, especially M2a macrophages that can promote muscle fibrosis via arginase-mediated metabolism. Aging of BMCs leads to a shift in myeloid cells in muscle toward the M2a phenotype that occurs independent of the age of the muscle in which the myeloid cells reside. This shift in macrophage phenotype can further promote muscle fibrosis during aging.

Our findings show that aging of the hematopoietic compartment is accompanied by an increase in the numbers of CD163+ M2a macrophages in skeletal muscle and show that the shift toward greater numbers of M2a macrophages is associated with increased muscle fibrosis. Furthermore, the data show that neither the increase in M2a macrophages nor muscle fibrosis occurs if the age-related decrease in muscle nNOS expression is prevented. Collectively, the results support the hypothesis that loss of NO production in aging muscle enables an increase of M2a macrophages that can promote fibrosis through arginase-dependent mechanisms (Fig.5). This resembles the mechanism that drives fibrosis in *mdx* muscle, although the magnitude of increase in M2a macrophages in aging muscle is only ∼threefold between 12 and 24 months of age while the increase in dystrophic muscle at the peak of inflammation is several hundred-fold compared to wild-type muscle (Wehling *et al*., 2001). However, the magnitude of fibrosis during muscle aging is similarly less. The observations suggest that fibrosis of senescent muscle could reflect a low-grade, chronic inflammation that increases tissue fibrosis.

Aging is generally viewed as involving a systemic increase in pro-inflammatory, Th1 cytokines that are associated with promoting many physiological changes associated with aging (Krabbe *et al*., 2004). Prominent among these increases, elevations of levels of the pro-inflammatory cytokine tumor necrosis factor-alpha (TNFα) have been reported most consistently in numerous senescent tissues, including muscle and blood (Greiwe *et al*., 2001; Visser *et al*., 2002; Schaap *et al*., 2009). We similarly observed elevations of TNFα expression in all aging muscles that we examined and found those increases were accompanied by elevations in other potent, pro-inflammatory cytokines including IFNγ and IL1β. Although IFNγ and TNFα are strong activators of the pro-inflammatory, M1 macrophage phenotype, we observed that the numbers of CD163+ M2a macrophages more than doubled in aging muscle, indicating that macrophage activation to the M2 phenotype is most strongly driven in aging muscle. However, this preferential activation to the M2a phenotype was not closely reflected by increases in IL-4 or IL-13. In contrast, all muscles examined showed significant elevations of IL-10 expression, supporting previous observations that IL-10 plays a central role in regulating M2a macrophage phenotype in muscle (Villalta *et al*., 2011; Deng *et al*., 2012). Although our findings clearly show preferential activation of macrophages to the M2a phenotype in muscle during aging, the finding is not entirely consistent with some previous observations. For example, comparisons of vastus lateralis biopsies from old (∼71 years) and young (∼32 years) humans showed no significant difference between the two groups in the number of CD163+ macrophages when the data were expressed as number of macrophages per muscle fiber (Przybyla *et al*., 2006). Nevertheless, increases in expression of IL-10 occurred in the aging, human muscles (Przybyla *et al*., 2006), similar to the present study. More recently, histological analysis of vastus lateralis biopsies of old and young human subjects also showed no significant difference in the number of CD68+ macrophages/fiber between the two groups (Tam *et al*., 2012). However, this latter investigation reported that mRNA expression levels of both CD68 and CD206, another marker of the M2 phenotype, approximately doubled in aged muscle, consistent with our current findings.

We were particularly interested in data obtained in our investigation that failed to implicate other cell types in age-related shifts in muscle myogenesis and fibrosis. For example, recent findings indicating a significant role of SiglecF+ eosinophils in IL-4 mediated activation of FAPs to drive regeneration of young muscle following acute injury (Heredia *et al*., 2013) suggested the possibility that perturbations in eosinophil numbers or IL-4 expression in aging muscle could influence the shift of senescent muscle to a less myogenic phenotype. However, we found eosinophils were rare in muscles at both 12 and 24 months of age, their numbers did not change during aging, they were located primarily in the epimysium rather than within muscle fascicles, and IL-4 levels did not decline with aging. We also anticipated that numbers of FoxP3 Tregs within the muscle could decline during aging, in view of recent findings that Tregs promote muscle regeneration and reduce fibrosis in young muscle following injury (Burzyn *et al*., 2013). However, Treg numbers and FoxP3 expression were low and did not change during aging, showing that reductions in myogenic capacity and increases in muscle fibrosis during aging were not likely the consequence of reductions in Treg functions within the muscle, as can occur in young, injured muscle.

We were also surprised that we did not detect signs of increased activation of Wnt signaling that would reflect transitions of satellite cells from a myogenic phenotype to a fibrogenic phenotype. This phenotypic transition is driven by a switch from Notch signaling to Wnt signaling, reflected in a requisite increase in axin-2 transcription (Brack *et al*., 2007). Previous investigations showed that during mouse aging, this transition corresponded to both a reduction in the regenerative capacity of satellite cells and an increase in muscle fibrosis, which was reflected by an approximately twofold increase in axin-2 mRNA as mice aged from about 5 months to about 25 months (Brack *et al*., 2007). However, we observed no increase in axin-2 mRNA in muscle between 12 and 24 months of age, indicating that the increased occurrence of fibrosis during muscle aging did not require elevation of axin-2 expression during that interval.

Reductions in satellite cell numbers have also been associated with shifting the balance between myogenesis and fibrosis in aging muscle, although conclusions vary with regard to whether reductions in satellite cell numbers actually occur during aging. Part of the explanation for the discrepancy reflects the ages selected for comparison. For example, numbers of Pax7 expressing cells per mouse myofiber in extensor digitorum longus muscle significantly declined when comparing 3- to 6-month-old muscles to 18- to 27-month-old muscles, but there was no significant reduction when comparing 11- to 12-month-old muscles to 18- to 27-month-old muscles (Shefer *et al*., 2006). That observation is consistent with the findings in the present investigation, which show no changes in satellite cell number per unit volume of muscle occurred between 12 and 24 months of age in mice. However, we did observe that expression of the nNOS transgene increased Pax7 expression and produced a strong trend (*P* = 0.057) for increased numbers of satellite cells in 12-month-old muscle. This finding is consistent with previous studies which showed that muscle-derived NO and exogenous NO donors can increase satellite cell numbers in young mice (Anderson, 2000; Buono *et al*., 2012) and *in vitro* (Soltow *et al*., 2010). Furthermore, we observed that the NO-induced elevation of Pax7 expression in 12-month-old muscles coincided with increases in expression of Vangl2. Previous investigators have demonstrated that NO can promote satellite cell proliferation *in vitro* via an NO-Vangl2-cGMP pathway that requires increased transcription of Vangl2 (Buono *et al*., 2012). Curiously, we did not find evidence for NO–Vangl2 induction of satellite cell proliferation in 24-month-old muscles, and the increase in Pax7 expression that was evident at 12 months of age in transgenic mice returned to wild-type levels at 24 months of age. Previous assays of NO induction of satellite cell proliferation *in vitro* showed a decline in the inductive effect in satellite cells isolated from old muscle (Leiter & Anderson, 2010). That observation combined with our findings suggests that the defect in NO activation of proliferation in old satellite cells may result from a lack of NO induction of Vangl2 expression that is specific to old muscle.

Our observations that muscle aging is associated with an increase in intramuscular M2a macrophages and increased fibrosis led us to speculate that aging of the hematopoietic lineage cells from which the macrophages were derived could influence the amplification of intramuscular M2a macrophages, independent of the age of the host tissue. We tested this by transplanting young BMCs into young or old recipients and then assaying M2a macrophage numbers and extent of fibrosis 8 months later. Our finding that young BMT into young recipients yielded numbers of intramuscular M2a macrophages that did not differ from nontransplanted, age-matched controls showed that macrophages derived from transplanted BMCs retained their normal capacity to enter muscle. However, young BMT into old recipients showed numbers of M2a macrophages in muscle that matched the age of the donors, not the recipient. Furthermore, the age-related increases in collagen type 1 and type 3 content of muscle did not occur in the muscles of mice that received young BMT. That finding validates the positive relationship between changes of M2a macrophage prevalence and the extent of muscle fibrosis, but it also tells us that factors endogenous both to aging skeletal muscle and to aging hematopoietic lineage cells work in concert to influence the numbers of M2a macrophages in aging muscle.

Recent, important findings showing that muscle fibrosis is amplified by depleting satellite cells from muscle during aging (Fry *et al*., 2015) combined with observations that satellite cell-derived factors can modulate the activity of fibroblasts in muscle (Fry *et al*., 2014) indicate that cells from multiple lineages may interact to regulate muscle fibrosis during aging. Our findings that implicate cells of the hematopoietic lineage in muscle fibrosis show that cells derived from the bone marrow also participate in that regulatory network. Notably, the extent to which aging muscle fibrosis is promoted by satellite cell depletions (Fry *et al*., 2015) resembles the extent to which aging of hematopoietic lineage cells appears to contribute to muscle fibrosis, in the present study. Continuing studies are directed toward exploring the potential regulatory interactions between satellite cells and bone marrow-derived cells that regulate fibrosis and other age-related changes of muscle.

## Experimental procedures

A more detailed account of experimental procedures can be found in the online supplemental information accompanying this article.

### Animal treatments

Experiments involving animals were conducted according to the National Institutes of Health Guide for the Care and Use of Laboratory Animals were approved by the Institutional Animal Care and Use Committee. Muscles were collected from wild-type (C57 BL/6) mice or mice that expressed a muscle-specific transgene encoding nNOS (Wehling *et al*., 2001) at 12 or 24 months of age and then used for analysis. As shown in previous work (Samengo *et al*., 2012), nNOS expression in wild-type, mouse quadriceps muscle declines ∼50% at the mRNA level and ∼70% at the protein level between 12 and 24 months of age. Expression of the nNOS transgene increases nNOS protein expression in muscle by ∼50-fold over wild-type levels (Wehling *et al*., 2001). Muscles were analyzed at 10 or 12 months of age which corresponds to ∼38–43 years of age in humans (Flurkey *et al*., 2007) and at 20 or 24 months of age which corresponds to humans at ∼62–69 years of age (Flurkey *et al*., 2007). Survivorship of nonirradiated C57 BL/6 mice is greater than 80% at 24 months (Flurkey *et al*., 2007). Survivorship of irradiated C57 BL/6 mice is greater than 60% at 20 months of age (current investigation). We have shown that significant sarcopenia occurs between 10 and 20 months of age in irradiated mice (present investigation) and between 12 and 24 months of age in nonirradiated mice (Samengo *et al*., 2012 and present investigation).

Other, wild-type mice were used for heterochronic, bone marrow transplantation in which donor mice were 2-month-old female mice and recipient mice were 2- or 12-month-old male mice. Recipient mice were subjected to myeloablative irradiation prior to bone marrow transplantation. Eight months following bone marrow transplantation, muscles and blood were collected from recipient mice. Engraftment of transplanted cells was assessed by fluorescent *in situ* hybridization for X and Y chromosome markers (Kreatech, Leica Labs, Buffalo Grove, IL, USA) in leukocytes isolated from blood collected from each mouse. Isolated leukocytes were adhered to microscope slides, and XY immunolabeled cells and XX immunolabeled cells on each slide were counted. Chimerism was expressed as the number of XX cells/total cell number for each mouse.

### RNA isolation and quantitative PCR

Frozen muscles were homogenized, and RNA was isolated, cDNA generated and QPCR performed as described previously (Villalta *et al*., 2011). Primers used for QPCR to assay expression levels of reference genes are reported in Table S1.

### Immunohistochemistry

Frozen, cross sections of entire quadriceps muscles were cut and immunolabeled for CD68+ macrophages (anti-CD68), M2a macrophages (anti-CD163 and anti-arginase-1), regulatory T cells (anti-FoxP3), eosinophils (antimajor basic protein), satellite cells (anti-Pax7), and connective tissue proteins (anticollagen type I, type III, and type V). Stereological techniques were used to determine the number of select cells per unit volume of muscle or the volume fraction of muscle comprised of selected connective tissue proteins, as described previously (Wehling *et al*., 2001).

### Statistics

Data are presented as mean ± SEM. One-way analysis of variance was used to test whether differences between groups were significant at *P* < 0.05. Significant differences between groups were identified using Tukey’s *post hoc* test.

## Supporting Information

**Fig. S1** Schematic of experimental design for bone marrow transplantation experiments.

**Table S1** Primers used for QPCR. **Appendix S1** Experimental procedures.
