# Supplementary figures and images for: Increases of M2a macrophages and fibrosis in aging muscle are influenced by bone marrow aging and negatively regulated by muscle-derived nitric oxide
Source: Aging Cell. 2015 May 25;14(4):678–88. doi: 10.1111/acel.12350 (PMC4531081; doi:10.1111/acel.12350)

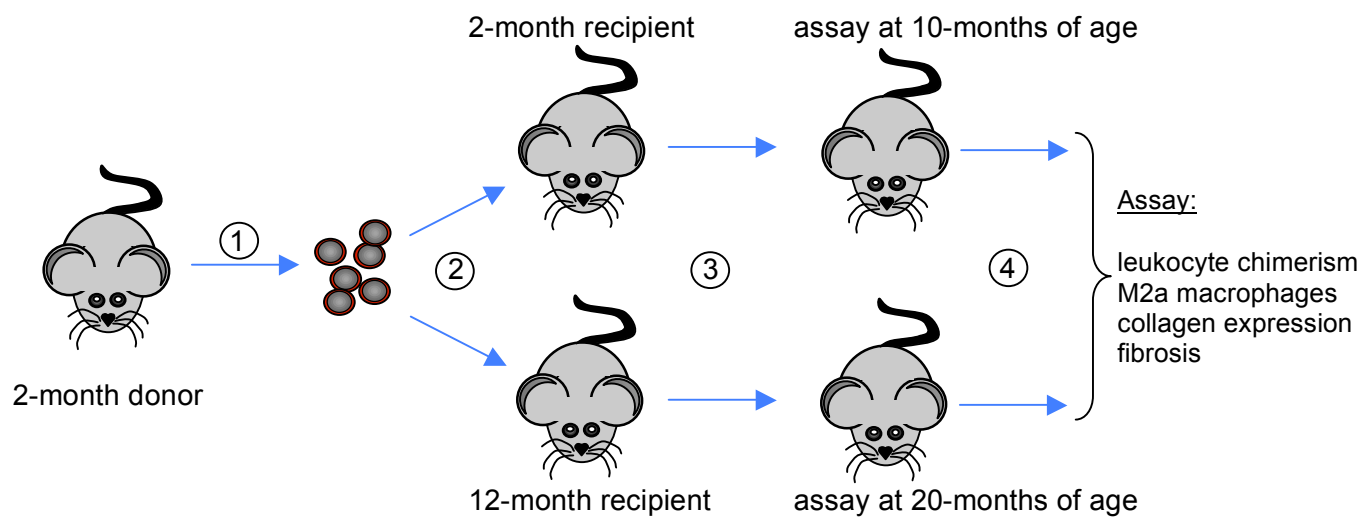

Supplement: Supplementary file 1 [file acel0014-0678-sd1.pdf]
