# Supplementary material for: Increases of M2a macrophages and fibrosis in aging muscle are influenced by bone marrow aging and negatively regulated by muscle-derived nitric oxide
Source: Aging Cell. 2015 May 25;14(4):678–88. doi: 10.1111/acel.12350 (PMC4531081; doi:10.1111/acel.12350)
Supplement: Supplementary file 2 [file acel0014-0678-sd2.docx]

Supplemental table.

| Gene | Accession Number |  | Direction (5’->3’) | Amplicon Size (bp) |
| --- | --- | --- | --- | --- |
| Col I | NM_007742.3 | Fwd | TGTGTGCGATGACGTGCAAT | 133 |
|  |  | Rev | GGGTCCCTCGACTCCTACA |  |
| Col III | NM_009930.2 | Fwd | ATCCCATTTGGAGAATGTTGTGC | 200 |
|  |  | Rev | GGACATGATTCACAGATTCCAGG |  |
| Col V | NM_016919.2 | Fwd | CGGGGTACTCCTGGTCCTAC | 118 |
|  |  | Rev | GCATCCCTACTTCCCCCTTG |  |
| TGFβ | NM_011577.1 | Fwd | CTCCACCTGCAAGACCAT | 84 |
|  |  | Rev | CTTAGTTTGGACAGGATCTGG |  |
| CD68 | NM_001291058.1 | Fwd | CAAAGCTTCTGCTGTGGAAAT | 140 |
|  |  | Rev | GACTGGTCACGGTTGCAAG |  |
| CD163 | NM_053094.2 | Fwd | GCAAAAACTGGCAGTGGG | 164 |
|  |  | Rev | GTCAAAATCACAGACGGAGC |  |
| TNFα | NM_013693.3 | Fwd | CTTCTGTCTACTGAACTTCGGG | 163 |
|  |  | Rev | CACTTGGTGGTTTGCTACGAC |  |
| IL6 | NM_031168.1 | Fwd | GAACAACGATGATGCACTTGC | 154 |
|  |  | Rev | CTTCATGTACTCCAGGTAGCTATGGT |  |
| IFNγ | NM_008337.3 | Fwd | GACAATCAGGCCATCAGCAAC | 161 |
|  |  | Rev | CGGATGAGCTCATTGAATGCTT |  |
| iNOS | NM_010927.3 | Fwd | CAGCACAGGAAATGTTTCAGC | 154 |
|  |  | Rev | TAGCCAGCGTACCGGATGA |  |
| IL13 | NM_008355.3 | Fwd | GTCCTGGCTCTTGCTTGC | 154 |
|  |  | Rev | CACTCCATACCATGCTGCC |  |
| IL4 | NM_021283.2 | Fwd | GGATGTGCCAAACGTCCTC | 126 |
|  |  | Rev | GAGTTCTTCTTCAAGCATGGAG |  |
| IL5 | NM_010558.1 | Fwd | CAAGCAATGAGACGATGAGG | 109 |
|  |  | Rev | CCACGGACAGTTTGATTCTTC |  |
| IL10 | NM_010548.2 | Fwd | CAAGGAGCATTTGAATTCCC | 157 |
|  |  | Rev | GGCCTTGTAGACACCTTGGTC |  |
| SiglecF | NM_145581.2 | Fwd | CAGCCCTGAAAGTAGCAGC | 159 |
|  |  | Rev | GTGGCTGTTCTTTCTGGGTC |  |
| FoxP3 | NM_054039.2 | Fwd | CTTTCACCTATGCCACCCTTATC | 77 |
|  |  | Rev | TAGATTTCATTGAGTGTCCTCTGC |  |
| Axin2 | NM_015732.4 | Fwd | GACGCACTGACCGACGATTC | 107 |
|  |  | Rev | CTGCGATGCATCTCTCTCTGG |  |
| Pax7 | NM_011039.2 | Fwd | CTCAGTGAGTTCGATTAGCCG | 144 |
|  |  | Rev | AGACGGTTCCCTTTGTCGC |  |
| Vangl2 | NM_033509.3 | Fwd | CCAAGTCCGTCCTGGCCAAG | 168 |
|  |  | Rev | GCTCATGCTCGGCTTCCTCG |  |
| Arg1 | NM_007482 | Fwd | CAATGAAGAGCTGGCTGGTGT | 153 |
|  |  | Rev | GTGTGAGCATCCACCCAAATG |  |
| RNPS1 | NM_001080127.1 | Fwd | AGGCTCACCAGGAATGTGAC | 196 |
|  |  | Rev | CTTGGCCATCAATTTGTCCT |  |
| SRP14 | NM_009273.4 | Fwd | GAGAGCGAGCAGTTCCTGAC | 196 |
|  |  | Rev | CGGTGCTGATCTTCCTTTTC |  |

Supplemental experimental procedures

*Mice.*

Wild-type mice (C57 BL/6) were obtained from Jackson Laboratories (Bar Harbor, ME, USA) or from the National Institute on Aging mouse colony. Mice expressing muscle specific transgenes for nNOS were produced as described previously (Wehling et al., 2001). Expression of the nNOS transgene was driven by the human skeletal muscle actin promoter (provided by Dr. Jeffrey Chamberlain, University of Washington). Rat nNOS cDNA was provided by Dr. James T. Stull (University of Texas, Southwestern). Transgenic mice were generated at the University of California, Irvine Transgenic Mouse Facility by microinjection of purified plasmids into zygotes from C57BL/6J × Balb C parents. F1 transgenic mice were crossed for at least 7 generations with C57BL/6J mice purchased from the Jackson Labs.

*Bone marrow transplantation.*

Male, 2-month-old, wild-type mice were given antibiotic-treated water containing 0.5 mg/ml trimethoprim/sulfamethoxazole for 6 days before irradiation. The mice then received total body irradiation of 950-1000R in one dose emanating from a cesium 137 source. 18 hours after irradiation, the mice were injected via tail vein with freshly isolated bone marrow cells (BMCs). BMCs for transplantation were flushed from tibias and femurs of 2-months-old female donor mice after euthanization by isoflurane inhalation. The femurs and tibias were sterilly dissected, cleaned and collected in sterile Petri dishes containing ice-cold sterile Dulbecco’s phosphate-buffered saline (DPBS; Gibco). BMCs were collected by flushing the bones with DPBS using a 23G needle. Red blood cells were lysed using 3 ml ACK lysing buffer (BioWhittaker) for 5 minutes on ice before reconstitution to 10 ml with DPBS. The cells were then filtered with a 70 µm cell strainer and then centrifuged at 500 g for 5 minutes. The cells were then washed and centrifuged two more times in DPBS to assure that non-cellular material was not transferred to the BMC transplant recipient. The final, cell pellet was re-suspended in DPBS at a concentration of 6.7 x 10^7^ cells / ml. Each recipient mouse was then injected with 150 μl containing 10^7^ cells via tail vein. The recipients were provided antibiotic water for 2 weeks after irradiation and then switched to acidified water without antibiotics.

*Chimerism assay.*

The engraftment of transplanted BMCs was evaluated using fluorescent in situ hybridization analysis for the X and Y chromosomes (XY-FISH). Blood samples were collected from the femoral artery at the time of dissection into tubes containing 100 μl of 20 mM EDTA ( pH 8.0). Red blood cells were lysed with 0.85% ammonium chloride for 10 min and centrifuged for 5 min at 800 g. The pellets were resuspended in 500 μl of 75 mM KCl and incubated for 1 minute at room temperature. Samples were then fixed with ice-cold 3:1 methanol and glacial acetic acid and centrifuged for 5 minutes at 1400 g. The pellets were resuspended in buffer, placed on slides and left to air dry. Slides were treated in 2X saline-sodium citrate (SSC) buffer with 0.6 M sodium chloride and 60 mM trisodium citrate (pH 7.0) at 37^o^ C for 15 min and then dehydrated in ethanol for 1 minute and air-dried. Slides were then treated with denaturing solution (70% formamide in 2X SSC (pH 7.0)) at 72^o^ C for 2 minutes and dehydrated in ice-cold 70%, 85% and 100% ethanol for 2 minutes each and air-dried. The X/Y chromosome probes (Kreatech) were denatured at 90^o^ C for 10 minutes. 2 μl of probe were applied to each slide and incubated overnight at 37^o^ C. After incubation, slides were washed successively in 0.4X SSC/ 0.3% Igepal at 72^o^ C for 2 minutes, 2X SSC/ 0.1% Igepal at room temperature for 1 minute, and 2X SSC at room temperature for 1 minute. Slides were then dehydrated in 70%, 85% and 100% ethanol for 1 minute each, air-dried and cover-slipped with Prolong Gold antifade reagent with DAPI (Invitrogen). The numbers of XY immunolabeled cells and XX immunolabeled cells on each slide were counted and chimerism was expressed as the number of XX cells / total cell number for each mouse.

*Production of Pax7 antibody and immunohistochemistry.*

Pax7 hybridoma cells were purchased from Developmental Studies Hybridoma Bank (Iowa City Iowa). Cells were cultured in complete medium consisting of DMEM with 1% penicillin- streptomycin (Gibco) and 20% heat-inactivated fetal bovine serum. After 48 hours, conditioned medium was collected and centrifuged at 400 g for 3 minutes. Supernatant was then affinity purified on a column containing anti-mouse IgG-agarose beads (Sigma-Aldrich) pretreated with Tris buffered saline (TBS) containing 150 mM NaCl, 50 mM Tris-HCl and 0.1% sodium azide. Bound protein was then eluted in 200 μL fractions in glycine elution buffer containing 100 mM glycine (ACROS Organics) and pH adjusted to 2.2 with HCl. The column was then washed with 10 volumes TBS. Protein concentration of each fraction was determined by testing absorbance at 280 nm. The fraction with highest protein concentration was used for immunohistochemistry. Specificity of the antibody was tested by western blotting with the antibody only or antibody pre-incubated with Pax7 blocking peptide (Aviva Systems Biology).

One quadriceps muscle from each mouse was dissected and rapidly frozen in isopentane cooled in liquid nitrogen. Frozen cross-sections were cut from the midbelly of each muscle at a thickness of 10 μm. The frozen sections were air-dried for 30 minutes and fixed in ice-cold acetone for 10 minutes, and endogenous peroxidase activity was quenched with 0.3% H_2_O_2_. Sections were then treated with blocking buffer from a mouse-on-mouse immunohistochemistry kit (M.O.M kit; Vector Laboratories) for 1 hour and immunolabeled with affinity purified mouse anti-Pax7 antibody (1/200) for 3 hours at room temperature. Sections were washed with 50 mM sodium phosphate buffer (pH 7.4) containing 200 mM sodium chloride (PBS) and then incubated with biotin-conjugated anti-mouse IgG (1/250) from the M.O.M kit for 30 minutes. Sections were subsequently washed with PBS and then incubated for 30 minutes with ABC reagents from the M.O.M kit. Staining was visualized with the peroxidase substrate 3-amino-9-ethylcarbazole (AEC kit; Vector Laboratories), yielding a red reaction product.

For immunohistochemistry for other antigens, acetone-fixed frozen sections of quadriceps were blocked in 3% bovine serum albumin (BSA) and 2% gelatin in 50 mM Tris buffer (pH 7.2) for 1 hour and then immunolabeled with rat anti-CD68 (1/100; Serotec), rabbit anti-CD163 (1/50; Santa Cruz Biotech), rabbit anti-major basic protein (1/3000; from Dr. James Lee, Mayo Clinic, Scottsdale), rabbit anti-collagen type I (1/50; Chemicon International) or goat anti-collagen type V (1/50; Southern Biotech) for 3 hours at room temperature or with rat anti-FoxP3 (1/10; eBioscience) or goat anti-collagen type III (1/50; Southern Biotech) overnight at 4^o^ C. Sections were washed with PBS and then probed with biotin-conjugated secondary antibodies (1/200; Vector Laboratories) for 30 minutes. Sections were subsequently washed with PBS and then incubated for 30 minutes with avidin D-conjugated horseradish peroxidase (1/1000; Vector Laboratories). Staining was visualized with the AEC kit.

The number of immunolabeled cells / volume of muscle tissue was determined by first measuring the total volume of each section using a stereological, point-counting technique to determine section area and then multiplying that value by the section thickness (10 μm) (Wehling et al., 2001). The numbers of immunolabeled cells in each section were counted and expressed as the number of cells / unit volume of each section. The volume fraction of muscle that was occupied by specific connective tissue proteins was determined by overlaying a 10 x 10 eyepiece micrometer over microscopic images of cross-sections of entire quadriceps muscle that were immunolabeled with antibodies to collagens type I, type III or type V. The percentage of grid intercepts that overlaid antibody-labeled connective tissue relative to total grid intercepts was determined to assess connective tissue volume fraction.

*Double-labeling for arginase-1 and CD163.*

Frozen, acetone-fixed sections of quadriceps muscles were treated with blocking buffer from M.O.M kit for 1 hour and then immunolabeled with a combination of mouse anti-arginase-1 (1/100; BD Transduction labs) and rabbit anti-CD163 (1/50; Santa Cruz Biotech) for 3 hours at room temperature. Sections were washed with PBS and then incubated with a combination of FITC conjugated anti-rabbit IgG and Texas Red conjugated anti-mouse IgG (1/100; Vector Laboratories) for 30 minutes in the dark. Sections were washed with PBS and cover-slipped with Prolong Gold anti-fade reagent with DAPI (Invitrogen).

*RNA isolation and quantitative PCR.*

Muscles were homogenized in Trizol (Invitrogen) and RNA extracted, isolated and DNase-treated using RNeasy spin columns according the manufacturer’s protocol (Qiagen). RNA was then electrophoresed on 1.2% agarose gels and RNA quality assessed by determining 28S and 18S ribosomal RNA integrity. Total RNA was reverse transcribed with Super Script Reverse Transcriptase II using oligo dTs to prime extension (Invitrogen). The cDNA was used to measure the expression of selected transcripts using SYBR green qPCR master mix according to the manufacturer’s protocol (Bio-Rad). Real-time PCR was performed on an iCycler thermocycler system equipped with iQ5 optical system software (Bio-Rad). We maximized the rigor of quantifying the relative levels of mRNA by following established guidelines for sample preparation, experimental design, data normalization and data analysis for QPCR (Nolan *et al.,* 2006; Bustin *et al*., 2009). Because expression of reference genes that are used to normalize QPCR data can vary between samples (Vandesompele *et al.,* 2002), we empirically identified reference genes that did not vary between our experimental groups using geNorm 3.5 software, as described previously (Villalta *et al.,* 2011). Based on that analysis, RNPS1 and SRP14 were validated as reference genes. The normalization factor for each sample was calculated by geometric averaging of the Ct values of both reference genes using the geNorm software. The highest relative expression for each gene was set to 1 and the other expression values were then scaled to that value. Primers used for QPCR are listed in Supplemental Table 1.

Supplemental figure legend.

Schematic of experimental design for bone marrow transplantation experiments. Bone marrow was isolated from female, 2-month-old wild-type mice (1). The isolated cells were then repeatedly washed in buffer and then transplanted to either 2-month-old or 12-month-old, wild-type, male recipients by tail vein injection (2). Mice in both groups were then housed for 8-months to permit bone marrow engraftment and aging (3). Tissues were collected for analysis when the young group was 10-months-old and contained 10-months-old bone-marrow-derived cells and the old group was 20-months-old containing 10-months-old bone-marrow-derived cells (4).
